# Supplementary material for: The epidemiologic and economic impact of a quadrivalent human papillomavirus vaccine in Thailand
Source: PLoS One. 2021 Feb 11;16(2):e0245894. doi: 10.1371/journal.pone.0245894 (PMC7877776; doi:10.1371/journal.pone.0245894)

# S1 Fig Estimated number of HPV-related disease events over 100 years in the routine 4vHPV vaccination, routine plus Catch-up 4vHPV Vaccination, compared to no vaccination (cervical cancer screening only) in a Thai population of 100,000 (Base Case Analysis)

# S1A Fig Estimated HPV 16/18 Related Incidence of Cervical Cancer Among Females over 100 Years

**
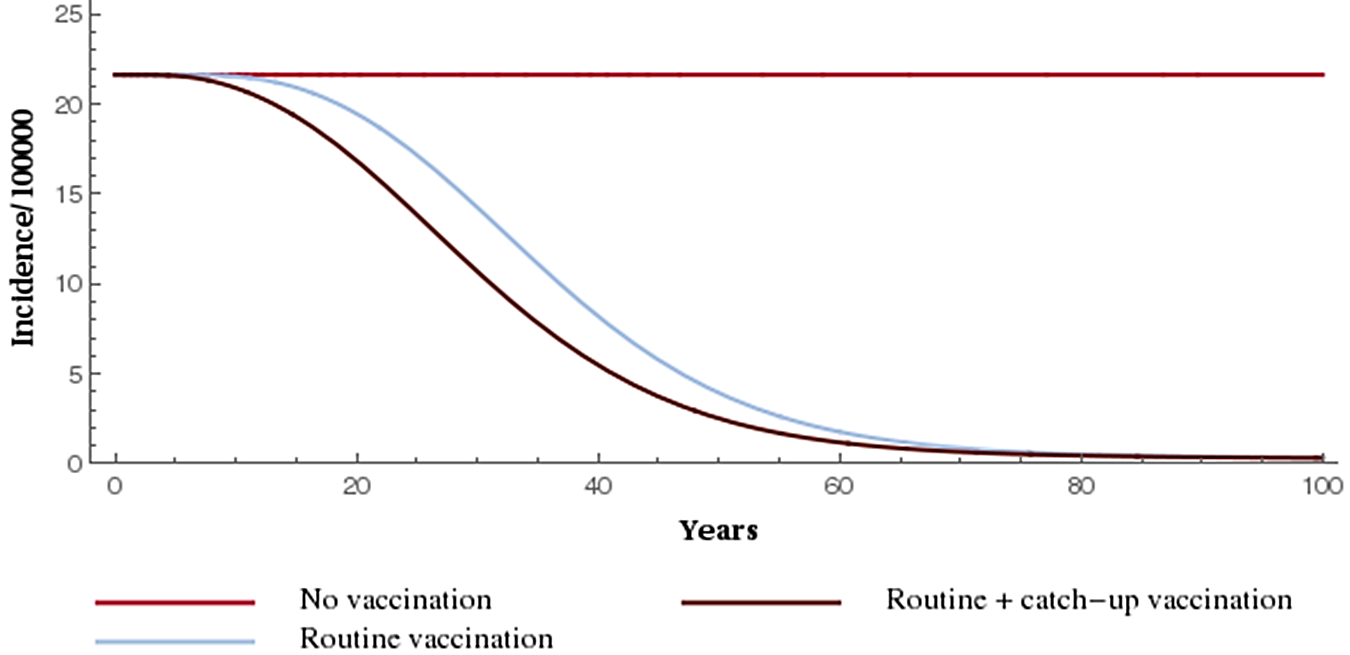
**

# S1B Fig Estimated HPV 16/18 Related Cervical Cancer Deaths Among Females over 100 years

**
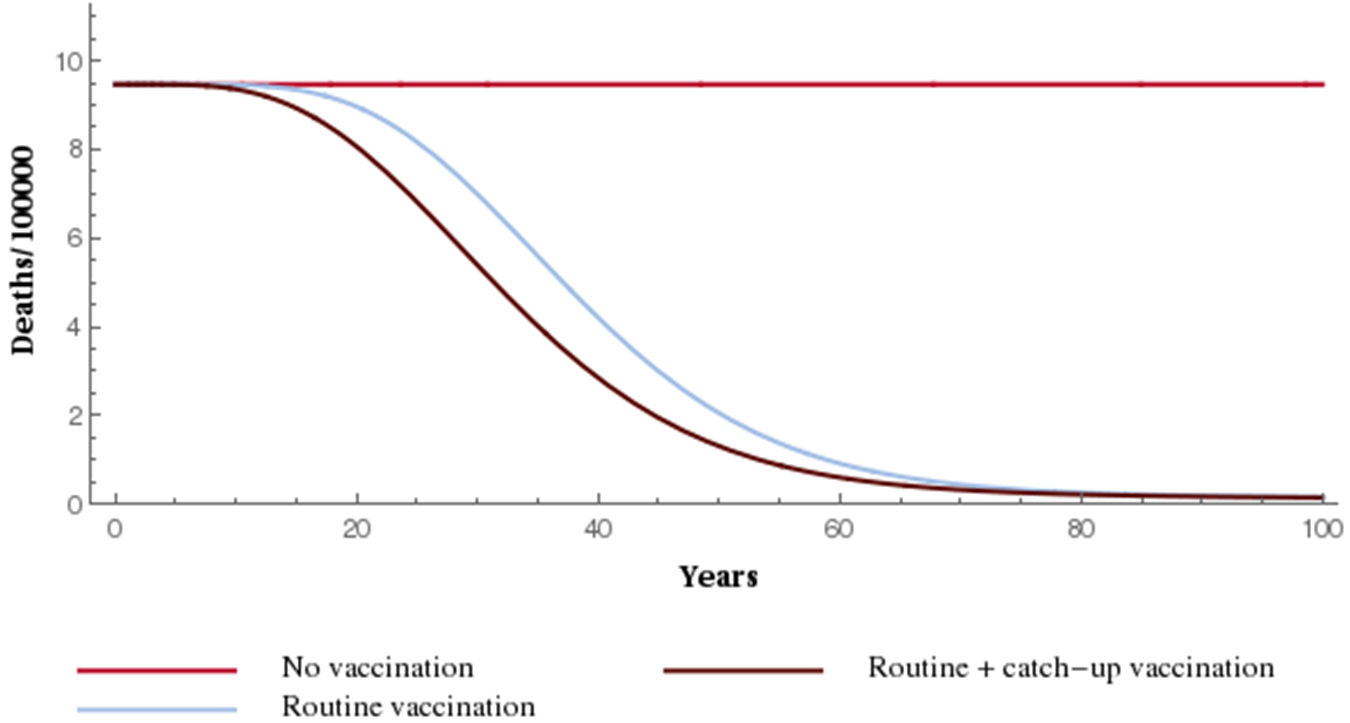
**

# S1C Fig Estimated HPV 16/18 Related incidence of CIN1 Among Females over 100 years


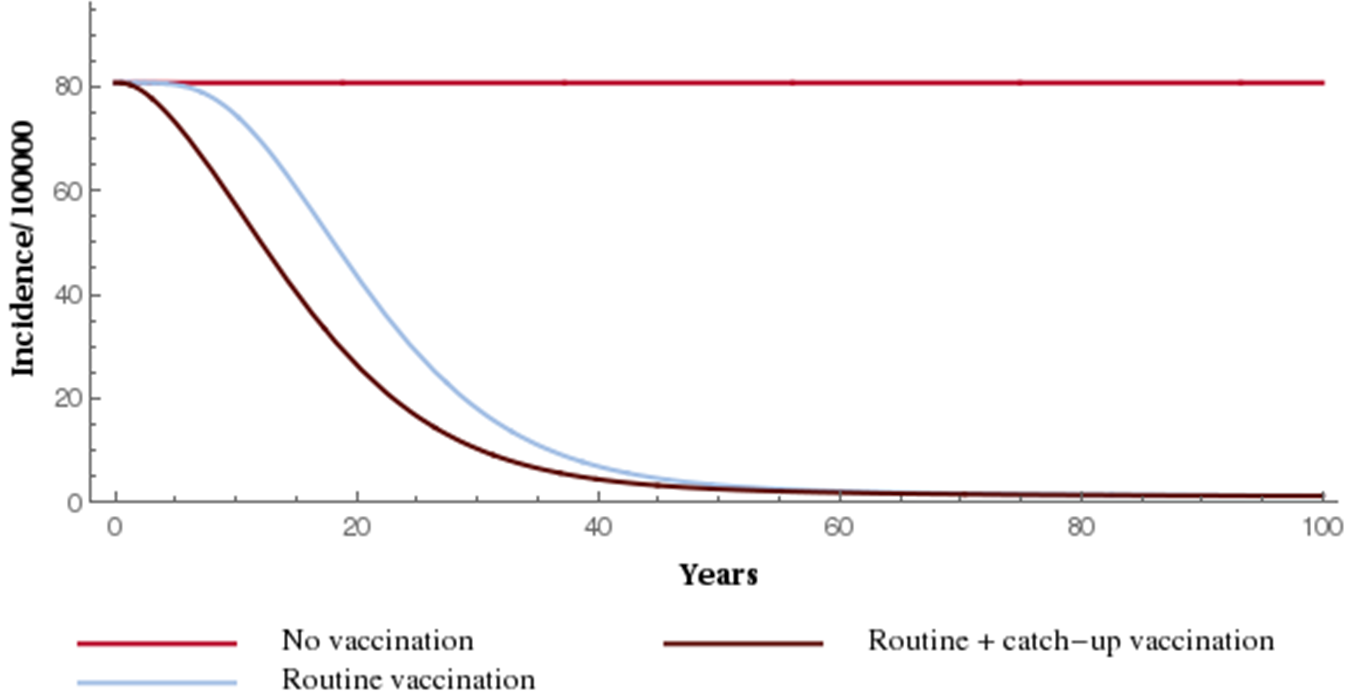


# S1D Fig Estimated HPV 16/18 Related Incidence of CIN 2/3 Among Females over 100 years


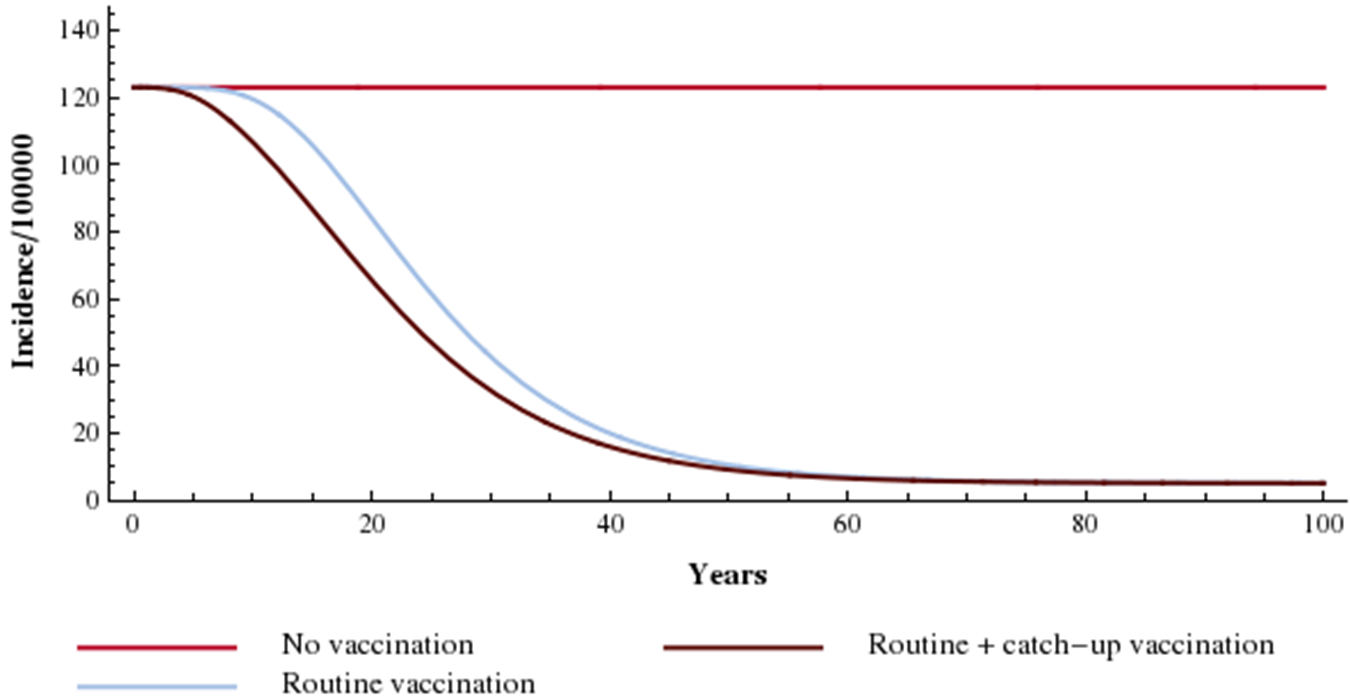


# S1E Fig Estimated HPV 6/11 Related Incidence of CIN1 Among Females over 100 years


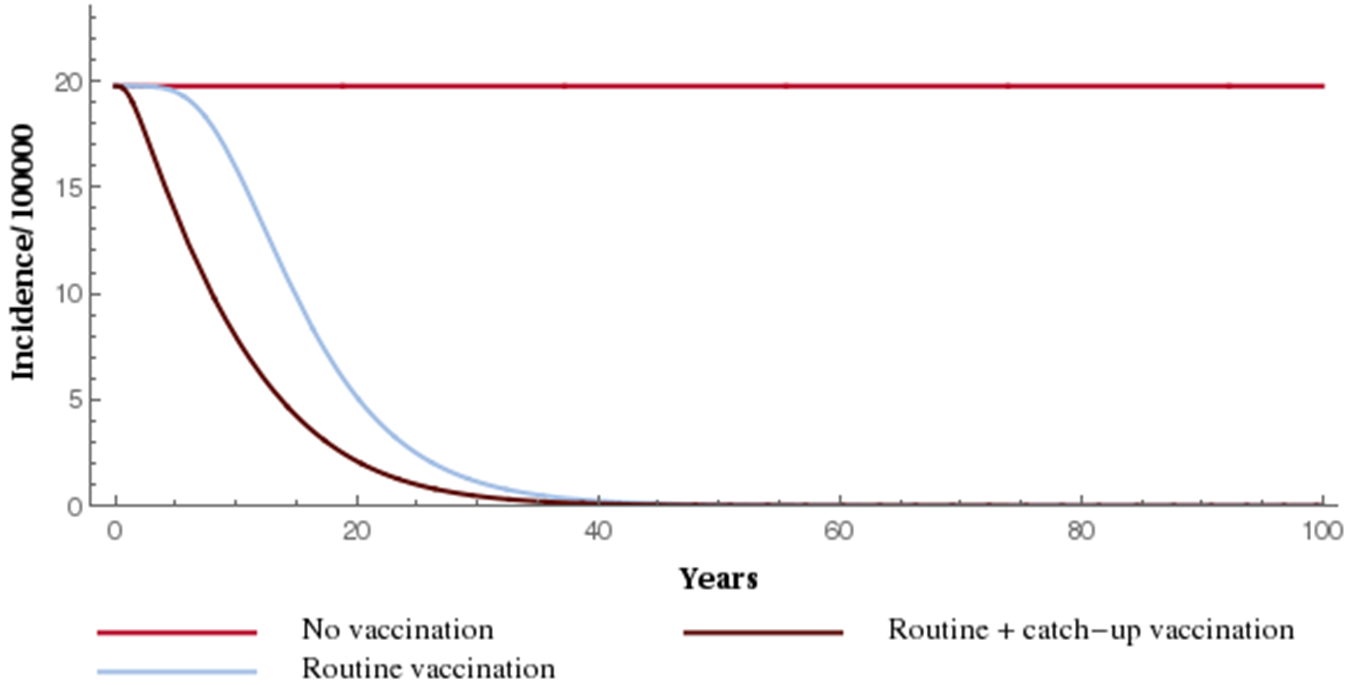


# S1F Fig Estimated HPV 6/11 Related Incidence of Genital Warts Among Females over 100 years


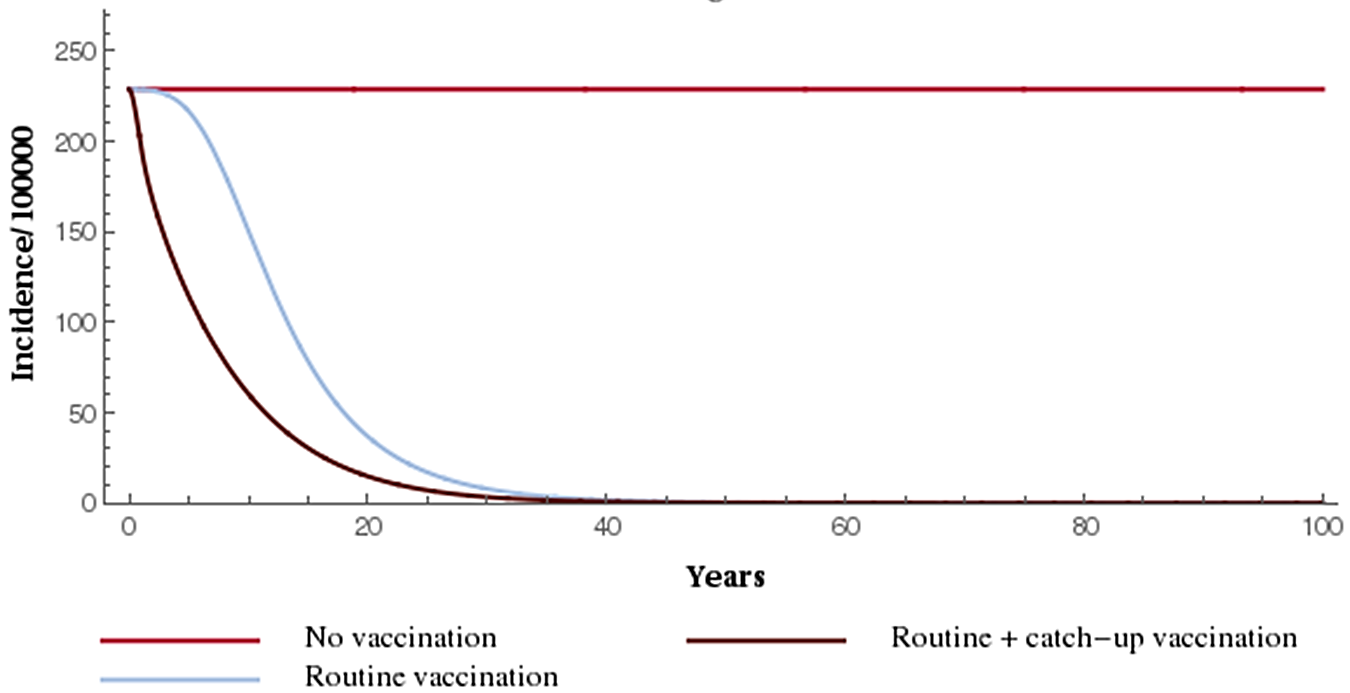

Supplement: S1 Fig — A, Estimated HPV 16/18 Related Incidence of Cervical Cancer Among Females over 100 Years; B, Estimated HPV 16/18 Related Cervical Cancer Deaths Among Females over 100 years; C, Estimated HPV 16/18 Related incidence of CIN1 Among Females over 100 years; D, Estimated HPV 16/18 Related Incidence of CIN 2/3 Among Females over 100 years; E, Estimated HPV 6/11 Related Incidence of CIN1 Among Females over 100 years; F, Estimated HPV 6/11 Related Incidence of Genital Warts Among Females over 100 years. (DOCX) [file pone.0245894.s001.docx]
