# Supplementary material for: The epidemiologic and economic impact of a quadrivalent human papillomavirus vaccine in Thailand
Source: PLoS One. 2021 Feb 11;16(2):e0245894. doi: 10.1371/journal.pone.0245894 (PMC7877776; doi:10.1371/journal.pone.0245894)

# S2 Fig Estimated HPV-related Treatment Costs avoided over 100 years by HPV Genotypes in Thailand when compared to no vaccination (screening only)

# S2A Fig. For routine 4vHPV vaccination


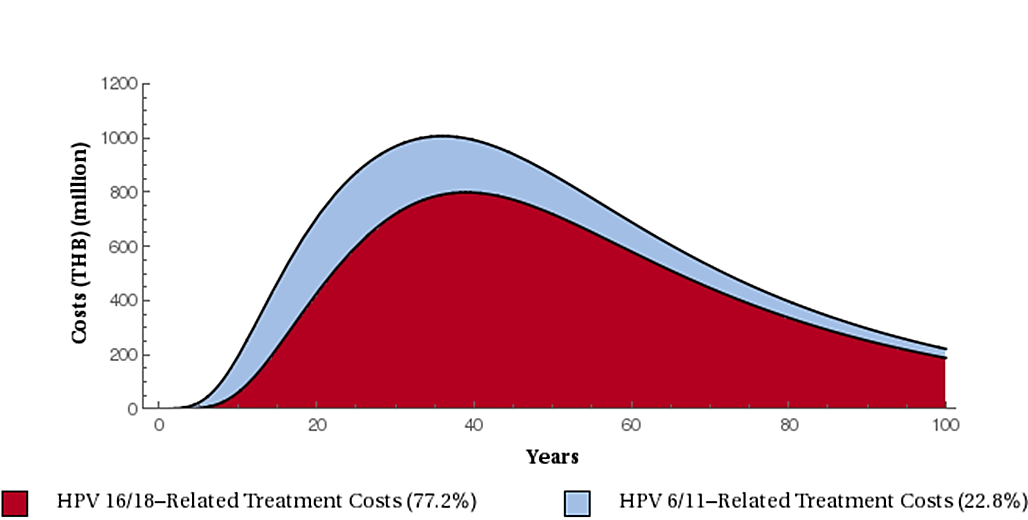


# S2B Fig. For routine plus catch-up 4vHPV vaccination


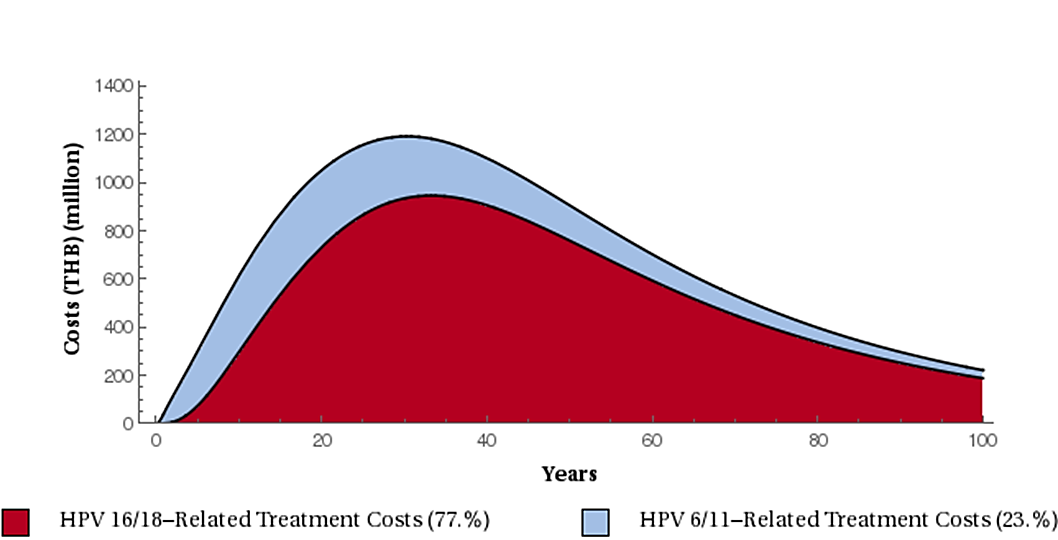

Supplement: S2 Fig — A, For routine 4vHPV vaccination; and B, For routine plus catch-up 4vHPV vaccination. (DOCX) [file pone.0245894.s002.docx]
