# Supplementary material for: The epidemiologic and economic impact of a quadrivalent human papillomavirus vaccine in Thailand
Source: PLoS One. 2021 Feb 11;16(2):e0245894. doi: 10.1371/journal.pone.0245894 (PMC7877776; doi:10.1371/journal.pone.0245894)
Supplement: S1 Table — (DOCX) [file pone.0245894.s003.docx]

# S1 Table. All-Cause Mortality and Cervical Cancer Mortality

| **Parameter** | **Estimate** | |  |
| --- | --- | --- | --- |
| **All-Cause Mortality Rate** | | | |
| **Age group** | **Females** | **Males** | **Source** |
| 0-4 years | 0.001 | 0.013 | WHO life table 2012 |
| 5-9 years | 0.001 | 0.002 |  |
| 10-14 years | 0.001 | 0.002 |  |
| 15-19 years | 0.002 | 0.006 |  |
| 20-24 years | 0.003 | 0.008 |  |
| 25-29 years | 0.005 | 0.010 |  |
| 30-34 years | 0.006 | 0.012 |  |
| 35-39 years | 0.008 | 0.018 |  |
| 40-44 years | 0.0101 | 0.024 |  |
| 45-49 years | 0.0131 | 0.028 |  |
| 50–54 years | 0.0192 | 0.038 |  |
| 55–59 years | 0.028 | 0.056 |  |
| 60–64 years | 0.046 | 0.087 |  |
| 65-69 years | 0.076 | 0.138 |  |
| 70+ years | 0.138 | 0.234 |  |
| **Age and Stage-Specific Cervical Cancer Mortality Rate (per year)** | | | |
| **LCC** | | | |
| **Age group** | **Female** | **Male** | **Source** |
| 15-29 years | 0.099 | - | Dasbach et al 2008[1] |
| 30-39 years | 0.041 | - |  |
| 40-49 years | 0.107 | - |  |
| 50-59 years | 0.1575 | - |  |
| 60-69 years | 0.33 | - |  |
| 70+ years | 0.182 | - |  |
| **RCC** | | | |
| **Age group** | **Female** | **Male** | **Source** |
| 15-29 years | 0.192 | - | Dasbach et al 2008 [1] |
| 30-39 years | 0.36 | - |  |
| 40-49 years | 0.388 | - |  |
| 50-59 years | 0.385 | - |  |
| 60-69 years | 0.4235 | - |  |
| 70+ years | 0.415 | - |  |
| **DCC** | | | |
| **Age group** | **Female** | **Male** | **Source** |
| 15-29 years | 0.412 | - | Dasbach et al 2008[1] |
| 30-39 years | 0.788 | - |  |
| 40-49 years | 0.5475 | - |  |
| 50-59 years | 0.6075 | - |  |
| 60-69 years | 0.7325 | - |  |
| 70+ years | 0.703 | - |  |

**Reference:**

1. Dasbach EJ, Insinga RP, Elbasha EH. The epidemiological and economic impact of a quadrivalent human papillomavirus vaccine (6/11/16/18) in the UK. BJOG : an international journal of obstetrics and gynaecology. 2008;115(8):947-56. Epub 2008/05/28. doi: 10.1111/j.1471-0528.2008.01743.x. PubMed PMID: 18503574.
