# Supplementary material for: The epidemiologic and economic impact of a quadrivalent human papillomavirus vaccine in Thailand
Source: PLoS One. 2021 Feb 11;16(2):e0245894. doi: 10.1371/journal.pone.0245894 (PMC7877776; doi:10.1371/journal.pone.0245894)
Supplement: S2 Table — (DOCX) [file pone.0245894.s004.docx]

# S2 Table. Sexual Behavior Parameters

| **Population annual mean number of sexual partners by sex and age group** | | | |
| --- | --- | --- | --- |
| **Age group** | **Females** | **Males** | **Source** |
| 10–14 years | 0.08 | 0.08 | Chompootaweep 1988 [1] |
| 15-19 years | 0.55 | 0.55 |  |
| 20-24 years | 2.3 | 2.3 |  |
| 25-29 years | 2.3 | 2.3 |  |
| 30-34 years | 2.3 | 2.3 |  |
| 35-39 years | 2.3 | 2.3 |  |
| 40–44 years | 0.9 | 0.9 |  |
| 45–49 years | 0.9 | 0.9 |  |
| 50–54 years | 0.9 | 0.9 |  |
| 55-59 years | 0.9 | 0.9 |  |
| 60-69 years | 0.8 | 0.8 |  |
| 70+ years | 0.8 | 0.8 |  |
| **Percent of the population in the following sexual activity risk groups** | | | |
| **Sexual Activity Groups** | **Females** | **Males** | **Source** |
| Low (Mean number of sexual partners per year: 0-1) | 31.3 | 25.6 | Tangmunkongvorakul et al 2011 [2];  Expert opinion |
| Medium (Mean number of sexual partners per year: 2-4) | 47.9 | 47.5 |  |
| High (Mean number of sexual partners per year: 5+) | 20.8 | 26.9 |  |

**Reference:**

1. Chompootaweep S. A Study of Reproductive Health in Adolescence of Secondary School Students and Teachers in Bangkok. Bangkok: Institute of Health Research, Chulalongkorn University. 1988.

2. Tangmunkongvorakul A, Carmichael G, Banwell C, Utomo ID, Sleigh A. Sexual perceptions and practices of young people in Northern Thailand. J Youth Stud. 2011;14(3):315-39. doi: 10.1080/13676261.2010.522562. PubMed PMID: 22319025.
