# Supplementary material for: The epidemiologic and economic impact of a quadrivalent human papillomavirus vaccine in Thailand
Source: PLoS One. 2021 Feb 11;16(2):e0245894. doi: 10.1371/journal.pone.0245894 (PMC7877776; doi:10.1371/journal.pone.0245894)
Supplement: S3 Table — (DOCX) [file pone.0245894.s005.docx]

**S3 Table.** Quality-of-Life Parameters

| **Health state** | **Utility value**  **(0-1)** | | **Source** |
| --- | --- | --- | --- |
| CIN 1 | 0.91 | | Myers et al. 2004 [1] |
| CIN 2/3, CIS | 0.87 | | Myers et al. 2004 [1] |
| Localized cervical cancer | 0.76 | | Myers et al. 2004 [1] |
| Regional cervical cancer | 0.67 | | Myers et al. 2004 [1] |
| Distant cervical cancer | 0.48 | | Gold et al. 1998 [2] |
| Cervical cancer survivor | 0.76 | | Gold et al. 1998 [2];  Andersen, 1996 [3];  Wenzel, 2005 [4] |
| Genital warts | 0.91 | | Myers et al. 2004 [1] |
| **No condition** | | | |
| **Age Group** | **Female** | **Male** | **Source** |
| 12–17 years | 0.930 | 0.930 | Golicki et al 2010 [5] |
| 18–24 years | 0.91 | 0.92 |  |
| 25–34 years | 0.91 | 0.92 |  |
| 35–44 years | 0.89 | 0.9 |  |
| 45–54 years | 0.86 | 0.87 |  |
| 55–64 years | 0.8 | 0.81 |  |
| >65 years | 0.78 | 0.76 |  |

**Reference:**

1. Myers E, Greenm S, Lipkus I. Patient preferences for health states related to HPV infection: visual analogue scales vs. time trade-off elicitation. Proceedings of the 21st International Papillomavirus Conference. Abstract no. 390.2. Mexico City, Mexico. 2004.

2. Gold MR, Franks P, McCoy KI, Fryback DG. Toward consistency in cost-utility analyses: using national measures to create condition-specific values. Medical care. 1998;36(6):778-92. Epub 1998/06/18. doi: 10.1097/00005650-199806000-00002. PubMed PMID: 9630120.

3. Andersen BL. Stress and quality of life following cervical cancer. Journal of the National Cancer Institute Monographs. 1996;(21):65-70. Epub 1996/01/01. PubMed PMID: 9023831.

4. Wenzel L, DeAlba I, Habbal R, Kluhsman BC, Fairclough D, Krebs LU, et al. Quality of life in long-term cervical cancer survivors. Gynecol Oncol. 2005;97(2):310-7. Epub 2005/05/03. doi: 10.1016/j.ygyno.2005.01.010. PubMed PMID: 15863123.

5. Golicki D, Jakubczyk M, Niewada M, Wrona W, Busschbach JJ. Valuation of EQ-5D health states in Poland: first TTO-based social value set in Central and Eastern Europe. Value in health : the journal of the International Society for Pharmacoeconomics and Outcomes Research. 2010;13(2):289-97. Epub 2009/09/12. doi: 10.1111/j.1524-4733.2009.00596.x. PubMed PMID: 19744296.
