# Supplementary material for: The epidemiologic and economic impact of a quadrivalent human papillomavirus vaccine in Thailand
Source: PLoS One. 2021 Feb 11;16(2):e0245894. doi: 10.1371/journal.pone.0245894 (PMC7877776; doi:10.1371/journal.pone.0245894)
Supplement: S4 Table — (DOCX) [file pone.0245894.s006.docx]

# S4 Table. Costs of diagnosing and treating HPV disease in Thailand (THB)^[[1]](#footnote-1)^

| **Cost category** | **Cost (THB)** | **Source** |
| --- | --- | --- |
| Vaccine (per each dose of quadrivalent HPV vaccine) | 500.00 | The cost proposed by The Ministry of Public Health of Thailand [1] [2] |
| Vaccine Administration | 0.00 | Family doctor |
| Genital warts treatment | 4215 | Termrungruanglert et al 2012 [3] |
| Cervical screening cytology* | 1876 | Sharma M, et al 2012 [4] |
| Colposcopy | 2253 | Sharma M, et al 2012 [4] |
| Biopsy | 642 | Sharma M, et al 2012 [4] |
| CIN 1 episode-of-care | 6036 | Termrungruanglert et al 2012 [3] |
| CIN 2 episode-of-care | 35619 | Termrungruanglert et al 2012 [3] |
| CIN 3 episode-of-care | 39494 | Termrungruanglert et al 2012 [3] |
| LCC treatment | 313135 | Termrungruanglert et al 2012 [3] |
| RCC treatment | 377858 | Termrungruanglert et al 2012 [3] |
| DCC treatment | 377858 | Termrungruanglert et al 2012 [3] |

*Cytology plus HPV test

**CIN = cervical intraepithelial neoplasia; DCC = distant cervical cancer; HPV = human papillomavirus; LCC =** localized cervical cancer; RCC = regional cervical cancer.

**Reference:**

1. Sajirawattanakul D, Krittin P. Govt urged to drop HPV vaccine plan. (Apr 09. 2012) (URL:<https://www.nationthailand.com/news/30179639>, Access 14 APR 2018). The Nation Thailand. 2012.

2. Ngorsuraches S, Nawanukool K, Petcharamanee K, Poopantrakool U. Parents' preferences and willingness-to-pay for human papilloma virus vaccines in Thailand. J Pharm Policy Pract. 2015;8(1):20-. doi: 10.1186/s40545-015-0040-8. PubMed PMID: 26199734.

3. Termrungruanglert W, Havanond P, Khemapech N, Lertmaharit S, Pongpanich S, Khorprasert C, et al. Cost and effectiveness evaluation of prophylactic HPV vaccine in developing countries. Value in health : the journal of the International Society for Pharmacoeconomics and Outcomes Research. 2012;15(1 Suppl):S29-34. Epub 2012/02/01. doi: 10.1016/j.jval.2011.11.007. PubMed PMID: 22265063.

4. Sharma M, Ortendahl J, van der Ham E, Sy S, Kim JJ. Cost-effectiveness of human papillomavirus vaccination and cervical cancer screening in Thailand. BJOG : an international journal of obstetrics and gynaecology. 2012;119(2):166-76. Epub 2011/04/13. doi: 10.1111/j.1471-0528.2011.02974.x. PubMed PMID: 21481160.

1. In the model the above cost values are rounded to the nearest Baht [↑](#footnote-ref-1)
